# Intraocular inflammation after intravitreal injection of aflibercept 8 mg for treatment-refractory neovascular age-related macular degeneration: a case report

**DOI:** 10.1186/s12886-024-03827-6

**Published:** 2025-01-23

**Authors:** Nozomu Hashiya, Ichiro Maruko, Yuri Miyaguchi, Ruka Maruko, Taiji Hasegawa, Tomohiro Iida

**Affiliations:** Department of Ophthalmology, Tokyo Women’s Medical University, 8-1 Kawadacho, Shinjuku-ku, Tokyo, 162-8666 Japan

**Keywords:** Aflibercept 8 mg, Intraocular inflammation, Retinal vasculitis, Retinal vascular occlusion, Treatment-refractory, Neovascular age-related macular degeneration

## Abstract

**Background:**

To report a case of intraocular inflammation (IOI) after intravitreal injection of aflibercept 8 mg for treatment-refractory neovascular age-related macular degeneration.

**Case presentation:**

An 80-year-old man with diabetes mellitus had neovascular age-related macular degeneration refractory to treatment with aflibercept 2 mg. Despite ten injections of faricimab, the exudation remained, and we switched to brolucizumab, which resulted in a mild IOI. The IOI improved with only topical steroids, and we switched back to aflibercept 2 mg for the exudation. However, the exudation remained, and we decided to switch to aflibercept 8 mg after careful discussion with the patient. Two weeks later, he experienced minor ocular pain and photophobia. One month later, although a dry macula was achieved, severe visual impairment occurred due to anterior chamber inflammation, retinal vasculitis, and retinal vascular occlusion. We diagnosed the severe IOI following aflibercept 8 mg and immediately started steroid eye drops and a sub-Tenon injection of triamcinolone acetonide. Although the inflammation resolved, his visual acuity did not improve.

**Conclusions:**

This case demonstrated a potential dose-dependent inflammatory response following aflibercept 8 mg, which did not occur with aflibercept 2 mg in patients with a history of intraocular inflammation.

## Background

Aflibercept 8 mg is a novel anti-vascular endothelial growth factor (VEGF) therapy recently approved in Japan to treat neovascular age-related macular degeneration (nAMD). Aflibercept 8 mg (0.07 ml) is a new anti-VEGF drug with a higher concentration and improved stability, allowing four times the intravitreal molar dose compared to the aflibercept 2 mg (0.05 ml). Since the introduction of brolucizumab and even faricimab, intraocular inflammation (IOI) after administration has been reported occasionally, and its safety has become a concern in recent years [1–5]. In the randomized, double-masked, non-inferiority phase 3 trial of aflibercept 8 mg in nAMD (PULSAR), aflibercept 8 mg and 2 mg had comparable safety profiles and a low incidence of IOI [6]. In the current study, we present a case of a patient with treatment-refractory nAMD who did not develop IOI with aflibercept 2 mg and developed IOI with perivascular hemorrhages and occlusive vasculitis after switching to aflibercept 8 mg.

## Case presentation

**Fig. 1:**
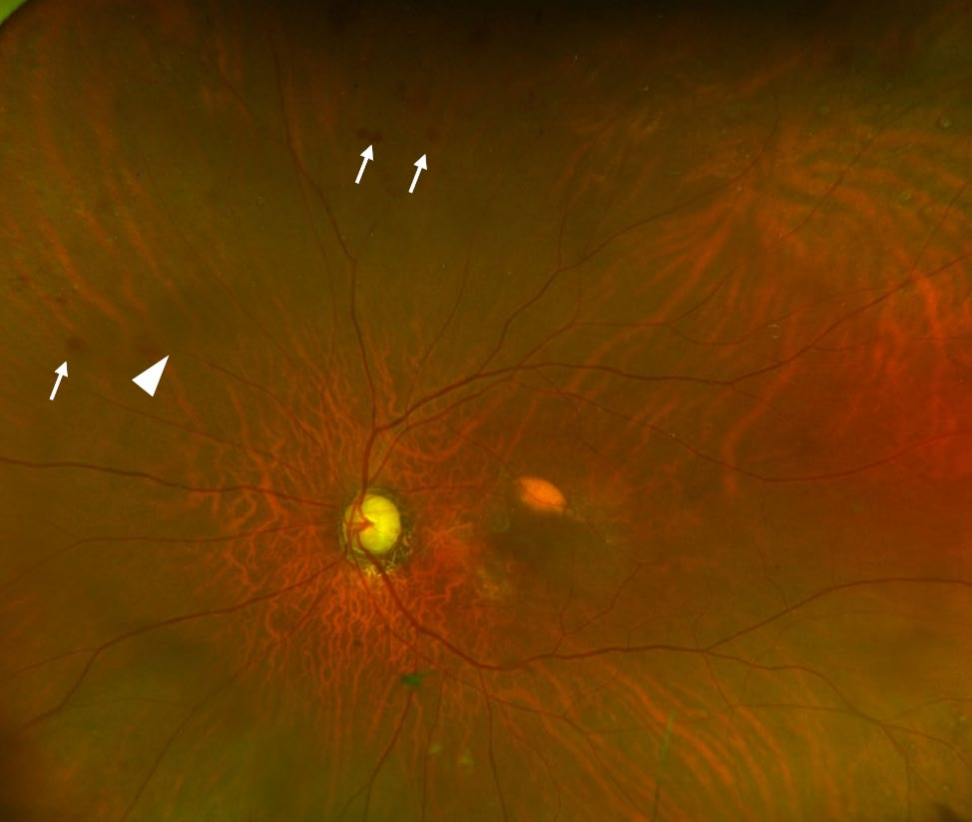
Fundus findings on ultra-widefield fundus photography after intravitreal injection of brolucizumab. Ultra-widefield fundus photography shows partial retinal vascular occlusion (white arrowhead) and perivascular hemorrhage (white arrows) without retinal vessel whitening, suggesting mild intraocular inflammation

**Fig. 2:**
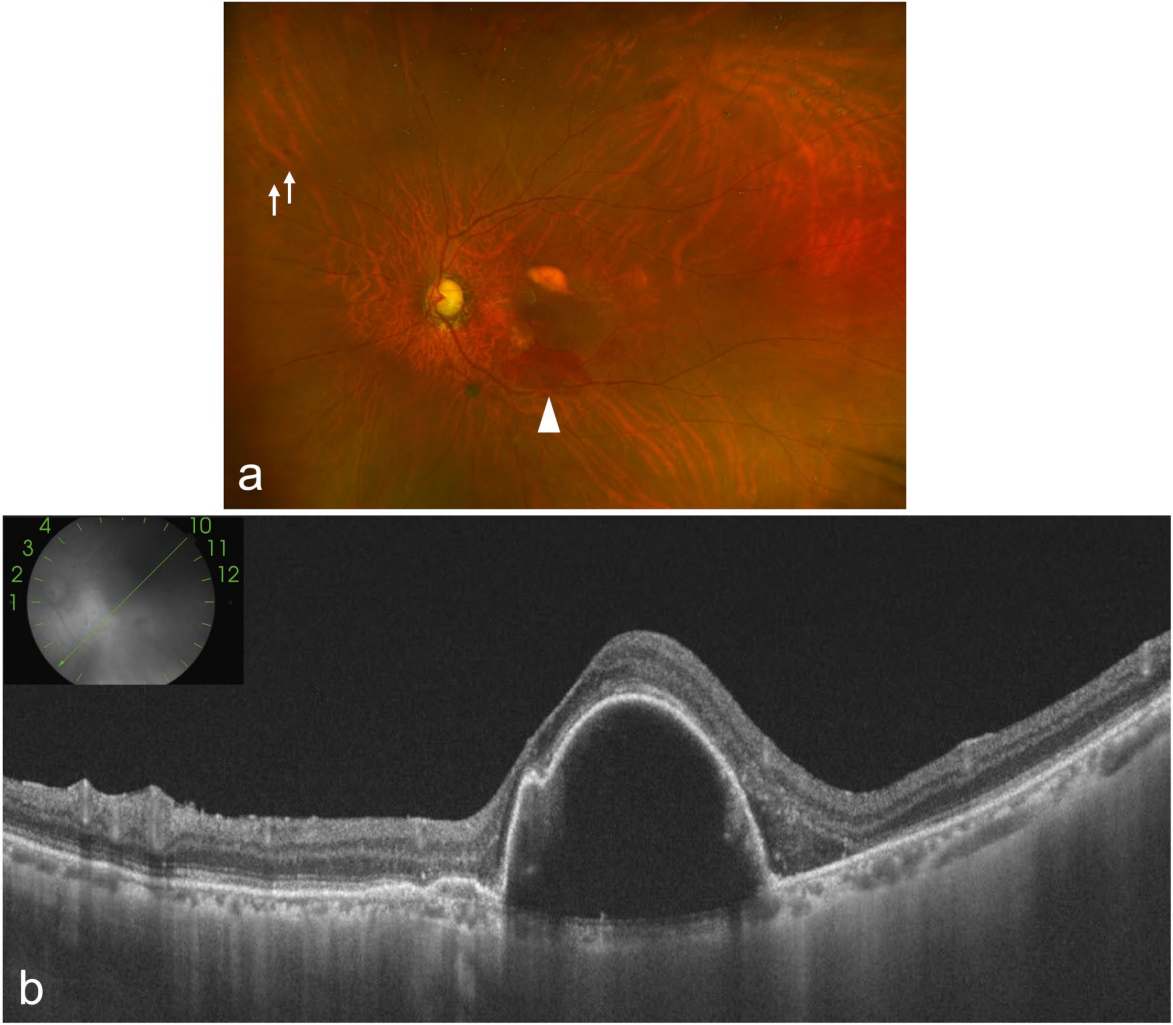
Baseline fundus findings on ultra-widefield fundus photography and optical coherence tomography (OCT) after aflibercept 2 mg and before aflibercept 8 mg. (**a**) Ultra-widefield fundus photography shows perivascular hemorrhage (white arrows) and subretinal hemorrhage (white arrowhead). (**b**) OCT of an oblique section demonstrated subretinal fluid (SRF) and a large sub-retinal pigment epithelium (RPE) fluid before intravitreal injection of aflibercept 8 mg

An 80-year-old Japanese male with a history of diabetes mellitus had previously undergone cataract surgery and was being treated for glaucoma and nAMD in his left eye. He was previously treated with aflibercept 2 mg for nAMD but was referred to our hospital because the macular exudation did not improve. At the initial visit, the best-corrected visual acuity (BCVA) of the left eye was 20/63, and optical coherence tomography (OCT) showed the subretinal fluid (SRF) and a large sub-retinal pigment epithelium (RPE) fluid, so the patient was diagnosed as refractory to aflibercept 2 mg, and treatment was switched to faricimab. Despite ten injections of faricimab, the BCVA worsened to 20/100, and a large sub-RPE remained. We therefore switched to brolucizumab. Following two injections of brolucizumab, a slit-lamp examination revealed anterior chamber cells (1+) and fine corneal precipitates without hypopyon and ultra-widefield color fundus images (Optos California, Nikon) demonstrated retinal vascular occlusion and perivascular hemorrhage at the nasal periphery, which suggested the occurrence of a mild non-infections IOI (Fig. 1). The inflammation was quickly resolved with only 0.1% betamethasone eye drops. Since SRF remained on OCT after IOI was under control, we switched back to aflibercept 2 mg, which resulted in further BCVA loss to 20/200. After switching back to aflibercept 2 mg, IOI was controlled, but subretinal hemorrhage worsened on Optos California (Fig. 2a), SRF remained on OCT (Fig. 2b), and BCVA further decreased to 20/200, indicating that macular exudation showed no improvement and remained refractory to treatment. Sixteen weeks after the final brolucizumab treatment and five weeks after switching to aflibercept 2 mg, when the IOI was confirmed to subside, we decided to switch to aflibercept 8 mg after careful discussion, expecting efficacy and safety.

**Fig. 3:**
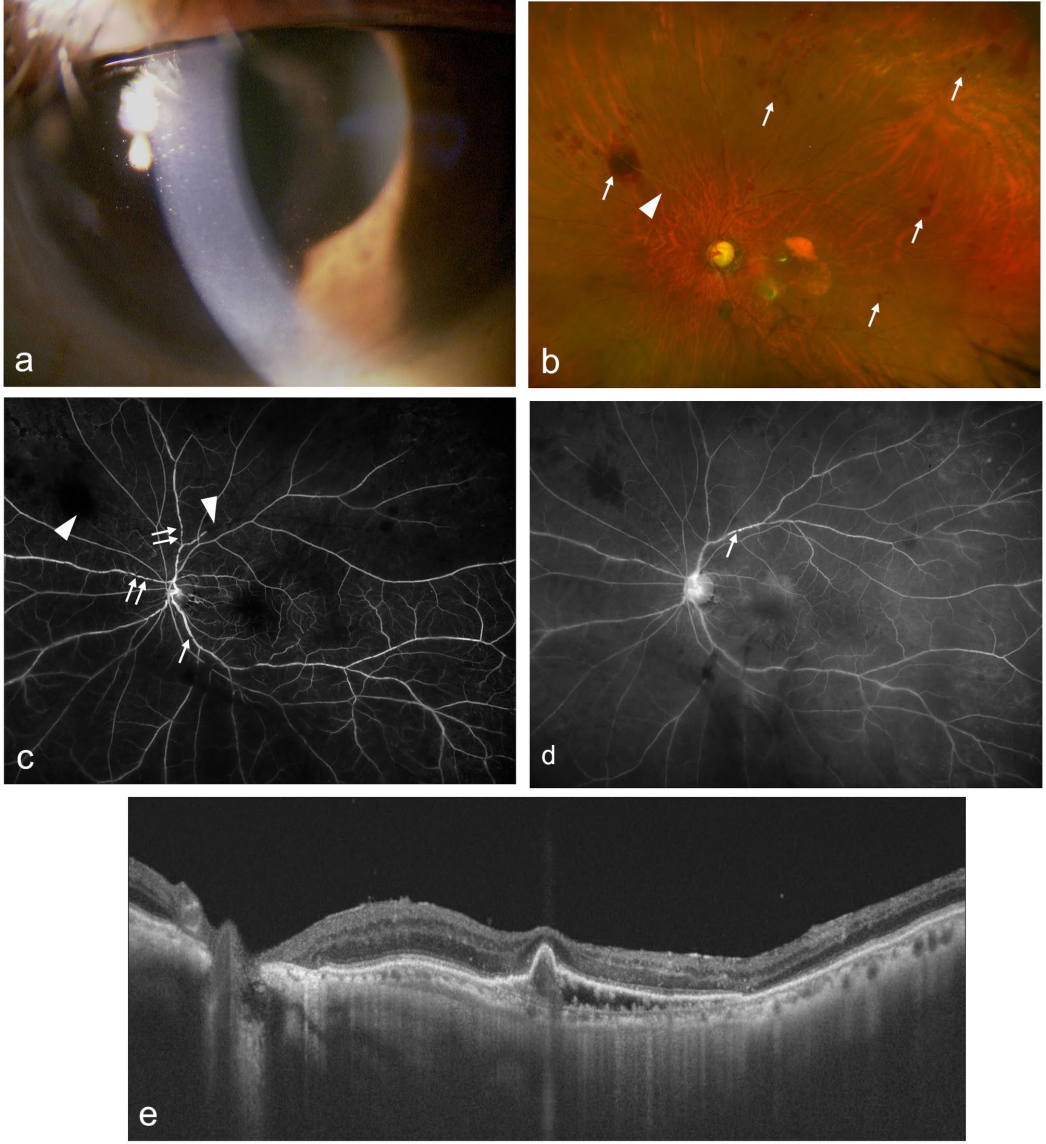
Slit-lamp examination and ultra-widefield fundus photography and ultra-widefield fluorescein angiography and optical coherence tomography (OCT) after intravitreal injection of aflibercept 8 mg. (**a**) Slit-lamp examination shows fine keratic precipitates and anterior chamber cells (1+) without hypopyon. (**b**) Ultra-widefield fundus photography shows whitening of retinal vessels at the nasal periphery (white arrowhead), and blot retinal hemorrhages around the peripheral vessels and vitreous opacities were also observed. (**c**) Ultra-widefield fluorescein angiography in the early phase showed a filling delay of peripheral retinal veins (white arrowheads) and multiple focal stenoses of retinal veins (white arrows). (**d**) Ultra-widefield fluorescein angiography in the late phase shows mild leakage from retinal veins and staining in the venous wall (white arrows). (**e**) OCT showed significant improvement, with the disappearance of SRF and a reduction in sub-RPE fluid size after intravitreal injection of aflibercept 8 mg

Two weeks later, he experienced minor ocular pain and photophobia but did not seek medical attention. At the one-month follow-up visit, BCVA decreased to 20/2000. Slit-lamp examination showed mild conjunctival injection, anterior chamber cells (1+), fine keratic precipitates (Fig. 3a), and vitreous cells (1+) without hypopyon. Ultra-widefield color fundus images showed whitening of retinal vessels on the nasal side of the optic nerve and blot retinal hemorrhages around these vessels (Fig. 3b). Ultra-widefield fluorescein angiography showed delayed filling of peripheral retinal veins, multiple retinal vein stenoses in the early phase (Fig. 3c), and mild retinal vein leakage in the late phase (Fig. 3d). We diagnosed the IOI following aflibercept 8 mg and immediately started steroid eye drops and a sub-Tenon injection of triamcinolone acetonide (20 mg/0.5 mL). The inflammation resolved, but his BCVA did not improve. Although IOI developed after aflibercept 8 mg, macular exudation, including large sub-RPE fluid, improved on OCT significantly (Fig. 3e). The dry macula could be maintained for eight weeks, but due to re-exudation, the patient was switched to ranibizumab after ten weeks, which settled down without inflammation.

## Discussion and conclusions

This report presents a case of intraocular inflammation associated with aflibercept 8 mg administration, accompanied by retinal vasculitis and retinal vascular occlusion, in a Japanese patient with treatment-refractory nAMD. The patient initially showed refractory to aflibercept 2 mg and faricimab and developed mild IOI when switched to brolucizumab. The inflammation was controlled with only 0.1% betamethasone eye drops and then switching back to aflibercept 2 mg. However, after switching to aflibercept 8 mg, more severe IOI developed, characterized by retinal hemorrhage and vascular occlusion. This case may reveal a potential dose-dependent relationship, where inflammation did not occur with aflibercept 2 mg but was triggered by the higher 8 mg dose.

Increasing the molar dose of intravitreal anti-VEGF therapy may enhance its efficacy and durability. It may result in an extension of dosing intervals and a reduction in the frequency of hospital visits. However, these benefits must be carefully considered against safety concerns, particularly those associated with higher doses. Clinical trials, such as the phase 2 cd study [7] and the phase 3 PULSAR study [6], have generally demonstrated comparable safety profiles between aflibercept 2 mg and 8 mg. For instance, the CANDELA trial reported only one case of mild iritis in the aflibercept 8 mg group, which resolved with topical treatment [7]. Similarly, in the PULSAR trial, although IOI occurred in both groups, were mild to moderate and did not require discontinuation of administration [6]. These results only show that severe IOI is infrequent but do not indicate that aflibercept 8 mg is without any risk at all. Calculations show that brolucizumab has the highest molar concentration of anti-VEGF currently available, followed by aflibercept 8 mg [8, 9]. It may not be a coincidence that this case showed intraocular inflammation with both drugs. However, there was no report on the IOI of aflibercept 8 mg in the US, even though it has already been approved by the Food and Drug Administration (FDA) in August 2023.

In Japanese populations, the incidence of IOI, particularly with brolucizumab, has been reported to be higher compared to global averages [2, 4]. Previous studies have shown IOI rates of 9–10% in Japanese nAMD patients treated with brolucizumab, with approximately one-third of these cases developing into retinal vasculitis or vascular occlusion [2]. Additionally, a one-year study found that a history of IOI and/or retinal occlusion, along with female gender, were significant risk factors for IOI after brolucizumab treatment [4]. These findings highlight the need for cautious patient selection, thorough education, and proactive management of inflammation when using brolucizumab or other anti-VEGF therapies in this demographic [10]. Recently there has been a report from Japan of an IOI after aflibercept 8 mg, where a small study in Japanese subjects reported IOI in 3 of 35 eyes (8.6%) treated with aflibercept 8 mg [11]. The limited sample size requires further investigation to determine whether the incidence is indeed elevated in the Japanese population. A larger multi-center analysis is needed better to understand the risk profile of aflibercept 8 mg, as higher rates of IOI have been observed with brolucizumab in Japan.

Since the introduction of brolucizumab and even faricimab, intraocular inflammation (IOI) after administration has been reported occasionally, raising safety concerns in recent years [1–5]. As shown in Table 1, brolucizumab has the highest molar concentration among anti-VEGF drugs, followed by aflibercept 8 mg and faricimab [12]. While earlier-generation anti-VEGF drugs had fewer reported IOI cases, the increasing reports with newer agents exhibiting higher molar concentrations of VEGF inhibitors suggest a potential dose-dependent inflammatory response to aflibercept 8 mg.

**Table 1:** Properties of anti-VEGF drugs

	Brolucizumab	Aflibercept 8 mg	Faricimab	Aflibercept 2 mg	Ranibizumab
Molecular weight	26 kDa	115 kDa	146 kDa	115 kDa	48 kDa
Equivalent molar dose	23.1	4.8	4	1.7	1

VEGF: Vascular Endothelial Growth Factor

This table summarizes the molecular weight and equivalent molar dose of anti-VEGF agents. Among these drugs, brolucizumab exhibits the highest molar concentration, followed by aflibercept 8 mg, faricimab, aflibercept 2 mg, and ranibizumab

Despite the occurrence of IOI, it is noteworthy that this patient sustained a dry macula for over two months, which is longer than previous treatments. This may suggest that aflibercept 8 mg may offer superior efficacy compared to aflibercept 2 mg, faricimab, and brolucizumab in the management of treatment-refractory nAMD. However, the potential dose-dependent inflammation requires a careful risk-benefit analysis, especially in patients with a history of ocular inflammation. Further research is needed to identify patient populations that may benefit from its enhanced efficacy without incurring significant inflammatory risks.

In conclusion, this case highlights the need for careful monitoring in patients with a history of IOI and suggests a potential for a dose-dependent inflammatory response to aflibercept 8 mg. When using high-dose anti-VEGF therapy, these considerations should be taken into account to optimize patient safety and minimize the risk of complications.

## Data Availability

No datasets were generated or analysed during the current study.

## Abbreviations

VEGF: Vascular endothelial growth factor
nAMD: Neovascular age-related macular degeneration
IOI: Intraocular inflammation
BCVA: Best-corrected visual acuity
OCT: Optical coherence tomography
SRF: Subretinal fluid
RPE: Retinal pigment epithelium
FDA: Food and Drug Administration
